# Updated Recommendations for Use of VariZIG — United States, 2013

**Published:** 2013-07-19

**Authors:** Mona Marin, Stephanie R. Bialek, Jane F. Seward

**Affiliations:** Div of Viral Diseases, National Center for Immunization and Respiratory Diseases, CDC

In December 2012, the Food and Drug Administration (FDA) approved VariZIG, a varicella zoster immune globulin preparation (Cangene Corporation, Winnipeg, Canada) for use in the United States for postexposure prophylaxis of varicella for persons at high risk for severe disease who lack evidence of immunity to varicella* and for whom varicella vaccine is contraindicated (1). Previously available under an investigational new drug (IND) expanded access protocol, VariZIG, a purified immune globulin preparation made from human plasma containing high levels of anti–varicella-zoster virus antibodies (immunoglobulin G), is the only varicella zoster immune globulin preparation currently available in the United States. VariZIG is now approved for administration as soon as possible following varicella-zoster virus exposure, ideally within 96 hours (4 days) for greatest effectiveness (2). CDC recommends administration of VariZIG as soon as possible after exposure to the varicella-zoster virus and within 10 days. CDC also has revised the patient groups recommended by the Advisory Committee on Immunization Practices (ACIP) to receive VariZIG by extending the period of eligibility for previously recommended premature infants from exposures to varicella-zoster virus during the neonatal period to exposures that occur during the entire period for which they require hospital care for their prematurity. The CDC recommendations for VariZIG use are now harmonized with the American Academy of Pediatrics (AAP) recommendations (3). This report summarizes data on the timing of administration of varicella zoster immune globulin in relation to exposure to varicella-zoster virus and provides the CDC updated recommendations for use of VariZIG that replace the 2007 ACIP recommendations.

## Background

Studies conducted in the late 1960s indicated that clinical varicella was prevented in susceptible, healthy children by administration of zoster immune globulin (ZIG) (prepared from patients recovering from herpes zoster) within 72 hours of household exposure (4). ZIG also lowered attack rates and modified disease severity among susceptible immunocompromised children when administered within 72 hours after household exposure (5,6). The definitions for susceptible children varied across studies and included children with negative or unknown history of varicella or those who were seronegative for varicella-zoster antibodies. The first commercial varicella zoster immune globulin preparation available in the United States, VZIG, was prepared from plasma obtained from healthy, volunteer blood donors identified by routine screening to have high antibody titers to varicella-zoster virus, and became available in 1978. Both serologic and clinical evaluations demonstrated that VZIG was equivalent to ZIG in preventing or modifying clinical illness in susceptible, immunocompromised children if administered within 96 hours of exposure to varicella (7,8). In a study of immunocompromised children who were administered VZIG within 96 hours of exposure, approximately one in five exposed children developed clinical varicella, and one in 20 developed subclinical disease compared with 65%–85% attack rates among historical controls (8). Among those in the study who became ill, the severity of clinical varicella (evaluated by percentage of patients with >100 lesions or with complications) was lower than expected on the basis of historic controls. The effectiveness of VZIG when administered >96 hours after initial exposure was not evaluated. Based on these findings and the licensure indications of the VZIG available in the United States, ACIP recommended VZIG for use within 96 hours of exposure (9). In February 2006, the VZIG supply was discontinued and a new product, VariZIG, became available under an IND protocol for administration within 96 hours of exposure (9,10).

## Methods

These recommendations reflect the ACIP work group discussions and review of scientific evidence related to use of varicella zoster immune globulin conducted during the development of the ACIP statements on prevention of varicella as well as a review of published literature to include reports with immune globulins with high anti–varicella-zoster virus antibodies used outside the United States >4 days after exposure to varicella-zoster virus. When data were not available, expert opinion was considered.

## Summary of Rationale for VariZIG Recommendations

### Timing of VariZIG administration

In May 2011, the FDA approved amendment of the IND protocol to extend the period for administration of VariZIG after exposure to varicella-zoster virus from 4 days (96 hours) to 10 days. Subsequently, in 2012, CDC published notification of FDA agreement with administration of investigational VariZIG as soon as possible after exposure and within 10 days (11). Limited experience from outside the United States with use of other immune globulin products with high levels of anti–varicella-zoster virus antibodies suggested that, compared with administration of the immune globulins within 4 days of exposure, administration >4 days (up to 10 days) after exposure resulted in comparable incidence of varicella and attenuation of disease (12–15). One study indicated an increase in varicella incidence with increasing time between exposure and administration of ZIG, but disease was attenuated in all cases (16). Considering these data, CDC recommends that VariZIG be administered as soon as possible after exposure and within 10 days. AAP also recommends administration of VariZIG within 10 days of exposure (3).

### Patient groups for whom VariZIG is recommended

In anticipation of availability of a licensed product for which the supply is projected to be adequate and to harmonize with recommendations from AAP, CDC revised the patient groups previously recommended by ACIP for use of VariZIG. The change refers to extending the period of eligibility for VariZIG administration for previously recommended premature infants from exposures to varicella-zoster virus during the neonatal period to exposures that occurred during the entire period for which they require hospital care for their prematurity. The risk for complications of postnatally acquired varicella in premature infants is unknown. Because the immune systems of premature infants (some of whom might be extremely low birthweight and spend months in neonatal intensive care units) might be compromised, they are considered, on the basis of expert opinion, at high risk for severe varicella; this increased risk is likely continued for as long as these infants remain hospitalized. Patients receiving monthly high-dose (≥400 mg/kg) immune globulin intravenous (IGIV) are likely to be protected and probably do not require VariZIG if the most recent dose of IGIV was administered ≤3 weeks before exposure (9).

## CDC Recommendations for Use of VariZIG

The decision to administer VariZIG depends on three factors: 1) whether the patient lacks evidence of immunity to varicella, 2) whether the exposure is likely to result in infection, and 3) whether the patient is at greater risk for varicella complications than the general population. For high-risk patients who have additional exposures to varicella-zoster virus ≥3 weeks after initial VariZIG administration, another dose of VariZIG should be considered.

### Timing of VariZIG administration

CDC recommends administration of VariZIG as soon as possible after exposure to varicella-zoster virus and within 10 days.

### Patient groups for whom VariZIG is recommended

Patients without evidence of immunity to varicella who are at high risk for severe varicella and complications, who have been exposed to varicella or herpes zoster, and for whom varicella vaccine is contraindicated, should receive VariZIG. Patient groups recommended by CDC to receive VariZIG include the following:

- Immunocompromised patients without evidence of immunity.
- Newborn infants whose mothers have signs and symptoms of varicella around the time of delivery (i.e., 5 days before to 2 days after).
- Hospitalized premature infants born at ≥28 weeks of gestation whose mothers do not have evidence of immunity to varicella.
- Hospitalized premature infants born at <28 weeks of gestation or who weigh ≤1,000 g at birth, regardless of their mothers’ evidence of immunity to varicella.
- Pregnant women without evidence of immunity.

## VariZIG Administration

VariZIG is supplied in 125-IU vials and should be administered intramuscularly as directed by the manufacturer. The recommended dose is 125 IU/10 kg of body weight, up to a maximum of 625 IU (five vials). The minimum dose is 62.5 IU (0.5 vial) for patients weighing ≤2.0 kg and 125 IU (one vial) for patients weighing 2.1–10.0 kg (2).

Unchanged from previous recommendations (9), for patients who become eligible for vaccination, varicella vaccine should be administered ≥5 months after VariZIG administration. Because varicella zoster immune globulin might prolong the incubation period by ≥1 week, any patient who receives VariZIG should be observed closely for signs and symptoms of varicella for 28 days after exposure. Antiviral therapy should be instituted immediately if signs or symptoms of varicella occur. Most common adverse reactions following VariZIG administration were pain at injection site (2%) and headache (2%) (2). Contraindications for VariZIG administration include a history of anaphylactic or severe systemic reactions to human immune globulins and IgA-deficient patients with antibodies against IgA and a history of hypersensitivity (2).

## How to Obtain VariZIG

VariZIG can be ordered from the exclusive U.S. distributor, FFF Enterprises (Temecula, California) (telephone, 800-843-7477; online at http://www.fffenterprises.com).

## Comment

The demand for VariZIG has declined significantly, commensurate with declining incidence of varicella (9). Nevertheless, exposures from varicella and from herpes zoster might still occur. Extending the time window for administration of VariZIG should increase availability of postexposure prophylaxis with VariZIG for persons at high risk for severe varicella. However, physicians are reminded that VariZIG should be administered as soon as possible following exposure. CDC recommendations for use of this product are now harmonized with those of AAP (3).

## Footnotes

* Evidence of immunity to varicella includes 1) documentation of age-appropriate vaccination with varicella vaccine, 2) laboratory evidence of immunity or laboratory confirmation of disease, 3) birth in the United States before 1980 (except for health-care personnel, pregnant women, and immunocompromised persons), or 4) health-care provider diagnosis or verification of a history of varicella or herpes zoster. For immunocompromised children aged 12 months to 6 years, 2 doses of varicella vaccine are considered age-appropriate vaccination.
